# Role of the SaeRS Two-Component Regulatory System in Group B *Streptococcus* Biofilm Formation on Human Fibrinogen

**DOI:** 10.3390/microorganisms12102096

**Published:** 2024-10-20

**Authors:** Francesco Coppolino, Alessia Berbiglia, Germana Lentini, Agata Famà, Giampiero Pietrocola, Giuseppe Teti, Concetta Beninati, Giuseppe Valerio De Gaetano

**Affiliations:** 1 Department of Human Pathology of Adult and Developmental Age “Gaetano Barresi”, University of Messina, 98168 Messina, Italy; 2 Department of Molecular Medicine, University of Pavia, 27100 Pavia, Italy; 3 Scylla Biotech S.r.l., 98168 Messina, Italy

**Keywords:** Group B *Streptococcus*, biofilm, two-component systems, extracellular matrix components

## Abstract

*Streptococcus agalactiae*, also known as Group B *Streptococcus* or GBS, is a commensal colonizer of human vaginal and gastrointestinal tracts that can also be a deadly pathogen for newborns, pregnant women, and the elderly. The SaeRS two-component regulatory system (TCS) positively regulates the expression of two GBS adhesins genes, but its role in the formation of biofilm, an important step in pathogenesis, has not been investigated. In the present study, we set up a novel model of GBS biofilm formation using surfaces coated with human fibrinogen (hFg). Biofilm mass and structure were analyzed by crystal violet staining and three-dimensional fluorescence microscopy, respectively. GBS growth on hFg resulted in the formation of a mature and abundant biofilm composed of bacterial cells and an extracellular matrix containing polysaccharides, proteins, and extracellular DNA (eDNA). Enzymatic and genetic analysis showed that GBS biofilm formation on hFg is dependent on proteins and eDNA in the extracellular matrix and on the presence of covalently linked cell wall proteins on the bacterial surface but not on the type-specific capsular polysaccharide. In the absence of the SaeR regulator of the SaeRS TCS, there was a significant reduction in biomass formation, with reduced numbers of bacterial cells, reduced eDNA content, and disruption of the biofilm architecture. Overall, our data suggest that GBS binding to hFg contributes to biofilm formation and that the SaeRS TCS plays an important role in this process.

## 1. Introduction

*Streptococcus agalactiae* (Group B *Streptococcus* or GBS) is an opportunistic Gram-positive human pathogen that colonizes the gastrointestinal and genitourinary tracts [1]. It is the causative agent of various infections, such as sepsis, pneumonia, and meningitis in neonates, pregnant women, and adults with underlying chronic illnesses and in the elderly [2,3]. Neonatal infections by GBS are grouped into two nosologically different categories. The early-onset disease (EOD) occurs in infants during the first few days following birth, while the late-onset disease (LOD) manifests from day 8 to 89 after delivery [4,5,6]. In the case of EOD, GBS is usually transmitted from the mother to the neonate during passage through the birth canal but can also reach the fetus after penetration of the fetal membranes [7]. In LOD, GBS can be acquired nosocomially via maternal breast milk or by horizontal transmission and is frequently associated with intestinal colonization [8]. According to the antigenic properties of the capsular polysaccharide, GBS can be classified into 10 serotypes, 5 of which (Ia, Ib, II, III, and V) account for almost the totality of human infections [1,9]. Serotype III GBS is are usually associated with neonatal meningitis, whereas serotype V GBS is largely responsible for invasive disease in adults [10,11,12]. GBS isolates are also classified into sequence types through the application of the multilocus sequence typing (MLST) technique [13].

Bacterial adherence is an important step in the process of host colonization and bacterial persistence. GBS is capable of interacting with different types of cells, such as epithelial and endothelial cells, by means of various surface-exposed proteins defined as adhesins [14]. GBS adhesins can interact with human extracellular matrix components (ECMs), such as fibrinogen (hFg), fibronectin, laminin, vitronectin, and plasminogen [15,16,17,18,19]. GBS can use various strategies to interact with human tissues in different host niches, and this versatility is reflected in the large set of adhesins that are dynamically expressed by GBS in different microenvironments. This is accomplished by sensing changes in the external environment and regulating gene expression through the activities of two-component regulatory systems (TCSs), which play major roles in bacterial virulence and antibiotic resistance [20,21]. TCSs are composed of a sensor histidine kinase (HK) and a response regulator (RR). The recognition of environmental stimuli by the extracellular domain of the HK triggers autophosphorylation in the HK cytoplasmic domain that phosphorylates, in turn, the receiver domain of the RR [22]. Phosphorylated RR binds to regulatory DNA sequences and promotes transcriptional activation or repression of different genes, many of which are involved in bacterial pathogenesis [23].

In many infections, biofilm assembly plays a prominent role by facilitating host colonization and bacterial persistence in the host. It is estimated that biofilms are associated with more than 80% of the infections involving the upper respiratory tract, the bloodstream, the urinary tract, and women’s genital tract [24,25,26]. Biofilms are microbial communities made up by one or more bacterial species that become enclosed within a self-produced matrix [27]. Their formation may occur on various body surfaces or medical devices such as heart valves, prostheses, and catheters [24]. Biofilm formation starts with the adhesion of planktonic bacteria to biotic or abiotic surfaces and continues with the progressive release of extracellular polymeric substances (EPSs) including proteins, polysaccharides, and extracellular DNA (eDNA). The majority of the *Streptococcaceae* family members have displayed the capability to assemble biofilms, with some studies focusing on GBS [28,29,30]. Acidic media, with pH values corresponding to those of vaginal secretions, promoted strong biofilm formation through a subset of type III strains belonging to the hypervirulent ST-17 lineage, which is often associated with invasive neonatal infection [31]. In another study, however, strains belonging to ST-17 and ST-19 lineages were weak biofilm formers compared to strains isolated from asymptomatic carriers [32]. Overall, different techniques have been used to induce biofilm formation by GBS with sometimes different results. Moreover, little is known of the molecular mechanisms involved in this process. A restricted number of studies have described the involvement of TCSs in the regulation of biofilm assembly by GBS [33,34]. Inactivation of the response regulator CovR in the GBS strain 2603 V/R has been shown to enhance biofilm formation on polystyrene plates [33]. Moreover, the TCS BceRS has been associated with an increased propensity of GBS to form biofilms and with increased bacterial protection from oxidative stress [34]. More information is available on the contribution of TCSs to biofilm formation with regard to other pathogens, including *Pseudomonas aeruginosa*, *Escherichia coli*, *Vibrio cholerae*, and *Staphylococcus aureus* [35,36]. Recently, a TCS homologous of the *S. aureus* virulence regulatory TCS SaeRS has been identified in GBS. SaeRS has been shown to become activated in vivo during GBS colonization and systemic infection and to promote the expression of two adhesins, the plasminogen-binding surface protein (PbsP) and the group B streptococcus vaginal adherence protein (BvaP), together with the *saeRS* operon [37,38,39,40]. Moreover, an *saeRS* knockout in GBS strain THN0901 (isolated from tilapia fish) showed that the mutant strain was deficient in biofilm formation, survived in fish blood, as well as showed virulence in an in vivo fish model and the mRNA expression of virulence factors [41].

In the present study, we set up a novel model of GBS biofilm formation involving surface coating with hFg, an extracellular matrix component (EMC) that is abundantly present at GBS colonization sites such as the intestinal and genital tracts. Moreover, we investigated whether SaeRS might be involved in biofilm assembly, given that this TCS was previously shown to play a role in staphylococcal biofilm formation [42,43]. We demonstrate that SaeRS inactivation significantly alters biofilm formation. These data may be useful for developing alternative strategies to prevent GBS infections by targeting the SaeRS TCS.

## 2. Materials and Methods

### 2.1. Bacterial Strains and Reagents

In this study, we used NEM316, a human GBS prototype strain belonging to sequence type 23 (ST23) and capsular type III, as well as the following mutant obtained in the NEM316 background. The Δ*cps* strain, a mutant lacking the capsular polysaccharide, and the Srt*A mutant, bearing a nonfunctional Sortase A enzyme, have been previously described [15,44]. The Δ*saeR* strain lacking the SaeR regulator was obtained and analyzed for genomic sequence as previously described [39]. None of the mutants showed differences in growth rates compared to the parental wild-type GBS strain during the log- and early-stationary phases. The *Lactococcus lactis* subsp. *cremoris* MG1363 strain was used as a control nonpathogenic organism. All strains were cultured in Todd Hewitt Broth (Difco, Becton Dickinson, Franklin Lake, NJ, USA; code 249240) supplemented with 5% (g/L) yeast extract (Becton Dickinson, Franklin Lake, NJ, USA; code 244020) at 37 °C. Human fibrinogen (hFg) was obtained from Fluka Analytical (Steinheim, Germany; code 46313), whereas human collagen type IV (hColl IV) was purchased from Sigma (Burlington, Ma, USA; code CC076).

### 2.2. Biofilm Assay on Extracellular Matrix Components

Twelve-well plates were coated with 1 µg/mL of hFg or Coll IV in phosphate-buffered saline (PBS: 137 mM NaCl; 2.7 mM KCl; 10 mM Na_2_HPO_4_; 1.8 mM KH_2_PO_4_) overnight at 4 °C. After washing with PBS (3 mL/well), GBS stock cultures were diluted 1:20 (5 × 10^6^ CFU/mL) in THY supplemented with 1% glucose (code 16325; Sigma, Burlington, Ma, USA) and 50 mM HEPES, pH = 7.8 (code H0887; Sigma, Burlington, MA, USA), and added to uncoated and hFg- or hColl IV-coated well plates. The plates were sealed to reduce oxygen exchange and left under shaking at 100 revolutions per minute (rpm) at 37 °C to prevent passive bacterial deposition. Following a 6 h incubation, the plates were gently washed three times to remove planktonic or loosely bound streptococci, and 2 mL of fresh medium was added to the wells. After 24 h at 37 °C under shaking conditions, the growth medium was removed again, and plates were carefully washed with PBS to remove unattached bacteria. After adding fresh medium to the washed wells, biofilm formation was assessed at 48 h. In some experiments, GBS biofilm formation was also induced by incubation with medium at 37 °C for 48 h without shaking. After the various incubations procedures, the wells were stained for 20 min at room temperature (RT) with 0.1% crystal violet (CV, code 94448; Sigma, Burlington, MA, USA) on a tilting incubator. After extensive washes to remove the unbound stain, the bound CV was solubilized by the addition of 30% acetic acid (code 45754, Fluka Analytical, Steinheim, Germany) in water before measuring the optical density at 596 nm (OD_596nm_) with a microplate reader. In each assay, uncoated wells without bacteria were further stained with CV to measure background CV absorbance, which was subtracted from the experimental values.

### 2.3. Fluorescence Microscopy

GBS biofilms grown on hFg or hColl IV were visualized with structured-illumination fluorescence microscopy using a Zeiss Observer microscope (serial number: 3834000913, Oberkochen, Germany) equipped with an Apotome apparatus. Images were acquired with AxionVision software 4.8.1, as previously described [45]. Briefly, GBS biofilms were grown as detailed above on coated coverslips for different periods (6, 24 and 48 h). Washed GBS biofilms were fixed with 3.7% formaldehyde (code: 119690025; Acros Organics, Geel, Antwerpen, Belgium) for 15 min at RT, and, after three extensive washes, 4′,6-diamidino-2-phenylindole (DAPI; D8417; Sigma, Burlington, MA, USA) stain was added to coverslips for 10 min at RT in the dark. Then, the samples were mounted and observed by using 1000× magnification. Live–dead staining was performed on unfixed 48-h-old biofilms grown on hFg by incubation with orange acridine (OA; code A-6014; Sigma, Burlington, MA, USA), a nucleic acid intercalating dye, at 50 ng/mL for 30 min at 37 °C in the dark under static conditions. Then, coverslips were carefully washed with PBS and allowed to dry. After mounting samples, pictures were taken with green and red filters to visualize viable and dead cells, respectively. In several experiments, COMSTAT software (v. 2.1) was used to evaluate the thickness and roughness of the three-dimensional biofilm acquired by fluorescence microscopy.

### 2.4. Enzymatic Treatment of GBS Biofilms

To explore the composition of the extracellular matrix, we used various enzymatic treatments. To test the role of eDNA, proteins and extracellular polysaccharides, 48-h-old washed biofilms were treated, respectively, with DNAse I (code D4513; Qiagen, Venlo, The Netherlands; 250 µg/mL), proteinase K (code 19133; Qiagen, Venlo, The Netherlands; 250 µg/mL), and β-N-acetylglucosaminidase (code A2264; Sigma, Burlington, MA, USA; 1 Units) for 2 h at 37 °C under static conditions. After all these three enzymatic treatments, the biofilms were gently washed and stained with CV. To microscopically visualize the effect of DNAse I on mature biofilms, DNAse-treated biofilms were washed, fixed, and stained with DAPI, as described above.

### 2.5. Viability Test

To investigate the bacterial viability in the biofilms assembled on hFg, colony-forming units (CFUs) were enumerated. To this end, 6- and 48-h-old biofilms were grown and, after several washes to remove unattached or loosely adherent bacteria, were statically treated with trypsin (1 mg/mL; Sigma, Burlington, MA, USA) for 10 min at 37 °C to release the bound bacteria, which were plated on agar plates. The rate of viable bacteria is indicated as CFU per biofilm.

### 2.6. Statistical Analysis

The statistical analysis of the differences in biofilm formation, as assessed by CV staining and in CFU values, was performed with GraphPad Prism 5 software. All experiments were repeated at least three times, and the data are expressed as means ± standard deviations (SDs). Differences between experimental groups were assessed for significance by the Mann–Whitney non-parametric test. A *p* value of 0.05 was used as the threshold for significance.

## 3. Results

### 3.1. GBS Forms Biofilms on Fibrinogen-Coated Surfaces

The most common in vitro protocol used to study GBS biofilm assembly involves growing GBS on uncoated surfaces under stationary conditions (“batch” mode) for periods of time ranging from several hours to a few days. It was shown, however, that growth under nonstationary conditions with medium exchange (“fed-batch” mode) can increase biomass production, with the added benefit of more closely mimicking the in vivo situation in which GBS is subjected to the shear forces of flowing mucosal secretions [31]. Moreover, the ability to strongly bind human fibrinogen (hFg), which is highly expressed on mucosal surfaces and in vaginal secretions [42,43,46,47,48], is a crucial property of invasive neonatal GBS isolates. For these reasons, in the present study, biofilm formation was studied under fed-batch conditions using hFg-coated plates. The biofilm formation on hFg by the NEM316 GBS strain was followed over the course of time by staining biomasses at early, intermediate, and late time points (6, 24, and 48 h, respectively).

**Figure 1:**
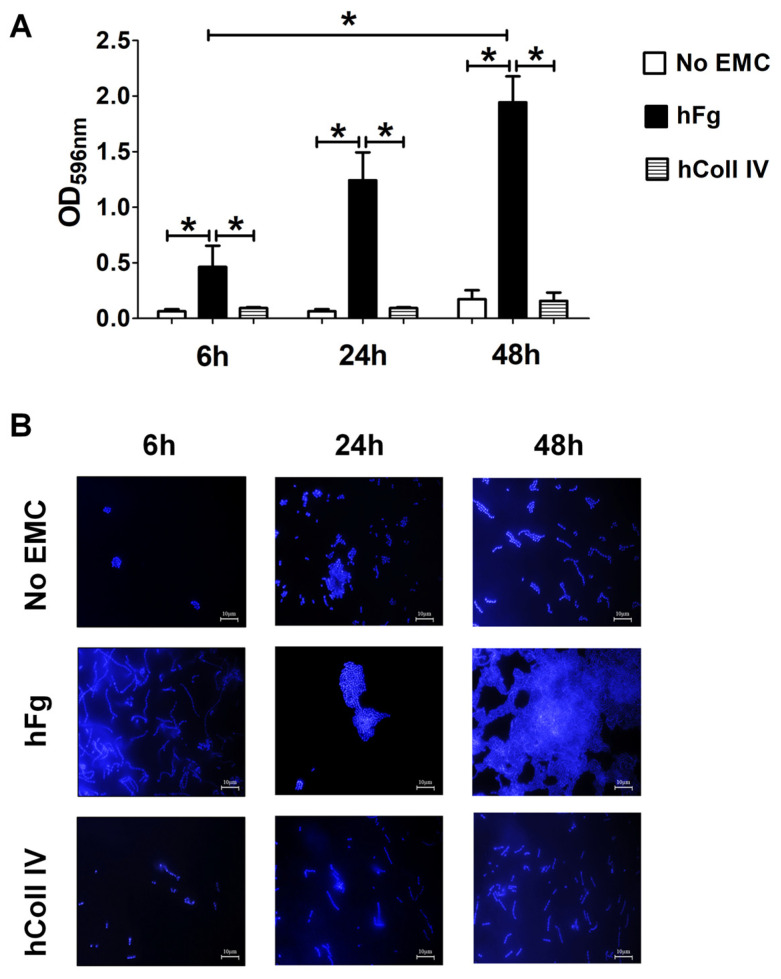
Biofilm formation by GBS on human fibrinogen. (**A**) Biofilm formation abilities of NEM316 wild-type (WT) strain on twelve-well plates coated with human fibrinogen (hFg) and collagen type IV (hColl IV) or without any extracellular matrix component (no EMC). GBS growth as biofilm was followed for 6, 24, and 48 h and verified with CV staining. Bacterial biomass was quantified by measuring the optical density at 596 nm (OD_596nm_). Results are means ± SD from three independent experiments performed in triplicate. * *p* < 0.05, as determined by Mann–Whitney statistical analysis. (**B**) Fluorescence microscopy analysis of GBS biofilm morphology over hFg. After growth in the conditions described in A, biofilms were stained with 4′,6-diamidino-2-phenylindole (DAPI, blue), which labeled DNA. In the absence of extracellular matrix components (no EMCs) or in the presence of hColl IV, only few and small scattered cell clusters or small aggregates are visible. In hFg-coated coverslips, a network of numerous, thick cell layers and shaped, rough biofilms are observed. Shown are representative images of three independent experiments. Scale bar = 10 µm.

Under these conditions, NEM316 produced considerably more biofilm on hFg-coated than on uncoated surfaces at all examined time points (Figure 1A). This property was specifically related to hFg since NEM316 failed to form robust biomasses on the plates coated with hColl IV (Figure 1A). In further studies, the ability of GBS to form biofilms on hFg was compared to that of *Lactococcus lactis*, a species that is unable to colonize or infect humans. However, *L. lactis* was totally unable to form biofilms on hFg, suggesting that this property may be specifically linked to the ability of GBS to associate with human tissues (Figure S1). Fluorescence microscopy analysis allowed for the further characterization of NEM316 sessile growth on hFg-coated surfaces. As depicted in Figure 1B, the number of bacteria was very low in uncoated or in hColl IV-coated well plates, with no recognizable organization of bacterial cells, with the exception of isolated, linearly arranged streptococcal chains. In particular, structured multilayered aggregates or multicolony three-dimensional aggregates were missing in uncoated or in hColl IV-coated well plates. Instead, in the presence of hFg, GBS rapidly gathered into abundant and compact multilayered biomasses with rounded shapes. In stark contrast, when the bacteria were grown under static conditions, no differences were noted in biomass formation irrespective of the presence of an hFg coating or on the expression of cell wall proteins. Moreover, equal amounts of biomass were formed by GBS and the non-human-associated control *L. lactis* strain (Figure S2). Taken together, these initial experiments showed how GBS can exploit surface-bound hFg to form progressively more robust, structured, and abundant biofilms. In the subsequent experiments, we assessed biofilm formation at 48 h, since the maximal biomass levels were reached at this time point.

### 3.2. Effect of SaeR Deletion on GBS Biofilm Formation

**Figure 2:**
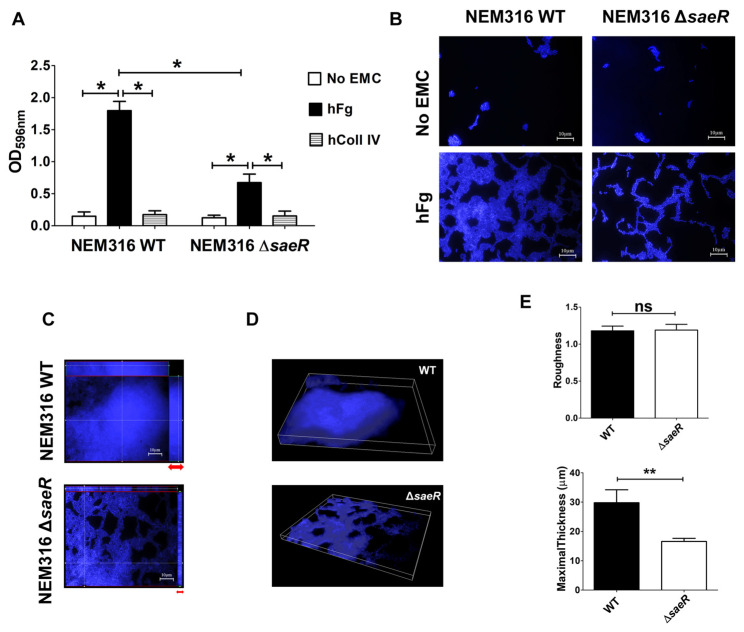
SaeR regulates GBS biofilm formation over human fibrinogen. (**A**) Biofilm formation abilities of NEM316 wild-type (WT) and NEM316 deleted for *saeR* (Δ*saeR*) strains on twelve-well plates coated with human fibrinogen (hFg) and human collagen type IV (hColl IV) or without any extracellular matrix components (no EMCs). GBS growth as biofilm was analyzed at 48 h and verified with CV staining. Bacterial biomass was quantified by measuring the optical density at 596 nm (OD_596nm_). Results are means ± SD from three independent experiments performed in triplicate. * *p* < 0.05, as determined by Mann–Whitney statistical analysis. (**B**) Morphological differences among GBS biofilms on hFg. WT and Δ*saeR* biofilms on hFg or uncoated wells (no EMCs) were visualized with 4′,6-diamidino-2-phenylindole (DAPI, blue). Shown are representative images of three independent experiments. Scale bar = 10 µm. (**C**) Assembly of bacteria in biofilm formed on hFg. *Z*-stack sections of 48-h-old GBS biofilms on hFg, with orthogonal views from *x*/*z* and *y*/*z* planes showing the distribution of bacterial genome stained with 4′,6-diamidino-2-phenylindole (DAPI, blue). Differences in biofilm height are indicated with red arrows. Shown are representative images of three independent experiments. Scale bar = 10 µm. (**D**) Three-dimensional reconstruction representative of hFg-biofilms formed by WT and Δ*saeR* mutant GBS strains after 48 h. Bacteria were stained with 4′,6-diamidino-2-phenylindole (DAPI, blue); (**E**) COMSTAT-2 quantification of maximal thickness (µm) and dimensionless roughness coefficient in biofilms formed on hFg at 48 h by WT and Δ*saeR* strains. ns, not significant; ** *p* < 0.01, as determined by Mann–Whitney statistical analysis. Data are from five different independent experiments.

It has been recently shown that the activation of the SaeRS TCS results in markedly increased GBS adherence to epithelial cells [39]. In order to study the functional role of SaeRS in biofilm formation, we used NEM316Δ*saeR*, a strain with a deletion in the gene encoding for the response regulator SaeR. Interestingly, the ability of GBS to form biomasses on hFg-coated but not hColl IV-coated surfaces was significantly, albeit partially, reduced in the absence of SaeR compared to that of the WT strain (Figure 2A). For example, in hFg biofilms, the average optical density after CV staining was ~1.8 for WT bacteria and ~0.7 for the Δ*saeR* mutant. The latter was not impaired in its ability to grow in suspension under standard laboratory conditions (Figure S3), ruling out reduced growth ability as a cause for reduced biofilm formation. Residual biofilm formation in the mutant suggests that other factors, in addition to SaeR, may be involved in biofilm formation on hFg-coated surfaces.

Next, we explored the appearance of the biofilms formed on hFg by the WT and the Δ*saeR* mutant using confocal microscopy. Microscopical examination showed that, in the absence of SaeR, GBS was unable to form compact biomasses on hFg, as suggested by the loosening of intercellular interactions in comparison with the WT strain (Figure 2B). Furthermore, while the WT bacteria showed considerable vertical growth, producing mushroom-shaped colonies, which are considered indicative of biofilm health and maturity [47], Δ*saeR* was comparatively impaired in its ability to grow as structured, multilayered aggregates. Overall, the WT bacteria showed many areas of dense association, suggesting bacterial multiplication in different planes, while this pattern was not observed with the Δ*saeR* mutant, as evidenced by orthogonal projections and three-dimensional analysis (Figure 2C, D). The measurement of biofilm thickness further evidenced the contribution of SaeR to the maintenance of biofilm architecture, while surface “roughness” was similar in the WT and the Δ*saeR* strain (Figure 2E).

**Figure 3:**
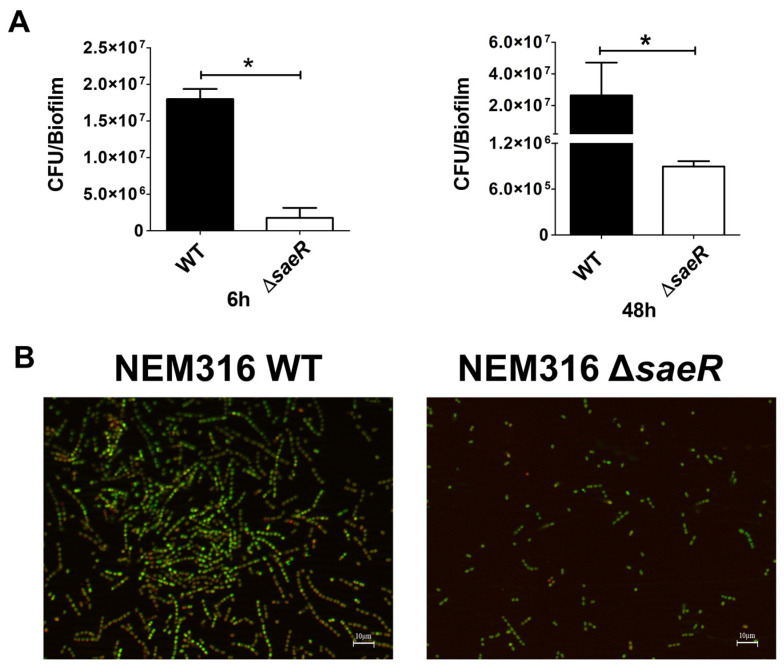
Streptococcal viability on biofilms formed on human fibrinogen. (**A**) Viability of NEM316 wild-type (WT) and NEM316 deleted for saeR (Δ*saeR*) strains on twelve-well plates coated with human fibrinogen (hFg). Biofilms at 48 h were detached with trypsin (1 mg/mL) and disaggregated by vigorously pipetting to enumerate colony forming units (CFUs) of WT and Δ*saeR* strains. Results are means ± SD from three independent experiments performed in triplicate. * *p* < 0.05, as determined by Mann–Whitney statistical analysis; (**B**) fluorescence images of 48-h-old GBS biofilms on hFg. WT and Δ*saeR* biofilms were incubated with orange acridine (OA), with viable and dead cells staining in green and red, respectively. Shown are representative images of three independent experiments. Scale bar = 10 µm.

In addition, we measured the number of viable bacteria in the WT and Δ*saeR* biofilms by CFU enumeration. The number of viable Δ*saeR* streptococci was lower than that of the WT bacteria at early and late time points during biofilm formation, suggesting that SaeR might be involved in the regulation of streptococcal viability (Figure 3A). The viability of Δ*saeR* and WT strains in 48 h old biofilms on hFg was further examined by fluorescence microscopy with orange acridine (OA), which differentially stains live (green) and dead (red) bacteria. Higher numbers of live bacteria in association with a sizeable portion of dead cells, which is a feature of mature biofilms, were detected in WT biofilms, compared with Δ*saeR* biofilms (Figure 3B). Moreover, the latter showed only rare dead bacteria, indicating that the absence of SaeR is associated with defective biofilm growth and maturation. Taken together, these data indicate that SaeR is involved in the formation of compact biomasses during sessile growth, as well as in biofilm maturation.

### 3.3. GBS Cell Wall Proteins Contribute to Biofilm Formation on hFg

**Figure 4:**
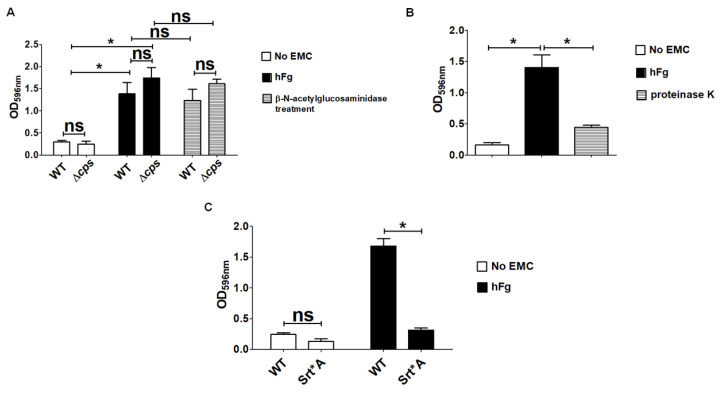
Features of extracellular polymeric matrix of GBS biofilm grown over human fibrinogen. (**A**) Analysis of the effects of β-N-acetylglucosaminidase (1 Units) on 48-h-old biofilms formed on human fibrinogen (hFg) by NEM316 wild-type (WT) strains and by two mutants with *saeR* and capsule deleted (Δ*saeR* and Δ*cps*, respectively). Results are means ± SD from three independent experiments performed in triplicate. ns, not significant; * *p* < 0.05, as determined by Mann–Whitney statistical analysis. (**B**) Analysis of proteinase K (250 µg/mL) in 48-h-old biofilms formed on human fibrinogen (hFg) by WT strain. Results are means ± SD from three independent experiments performed in triplicate. * *p* < 0.05, as determined by Mann–Whitney statistical analysis. (**C**) Role of SrtA-dependent cell wall proteins in GBS biofilm formation over hFg. Srt*A, mutant strain with non-functional SrtA. Bacterial biomass was quantified by measuring the optical density at 596 nm (OD_596nm_). Results are means ± SD from three independent experiments performed in triplicate. ns, not significant; * *p* < 0.05, as determined by Mann–Whitney statistical analysis.

The polysaccharidic capsule represents the most important virulence factor of GBS by virtue of its ability to prevent complement activation and to enable bacteria to evade phagocytic killing. Moreover, the capsular polysaccharide was shown to mediate the biofilm formation by GBS grown under static conditions in the presence of human plasma [48]. In light of this, we wondered if the GBS capsule could play a role in the biofilm formation on hFg. To this end, we compared a capsule-deleted mutant (Δ*cps*) with the parental WT strain for biofilm formation on hFg-coated surfaces. However, no differences between the Δ*cps* and the WT strain were noted in the stainable biomass assembled on hFg, suggesting that the capsule is not required for biofilm formation (Figure 4A). As biofilms contain a self-produced matrix containing numerous biomolecules, including polysaccharides and proteins, a common approach to study biofilm composition involves enzymatic treatments. Therefore, we treated the WT and Δ*cps* biofilms with the enzyme β-N-acetylglucosaminidase that cleaves β-(1,6)-linked poly *N*-acetyl glucosamine (PNAG), which is a major component of the exopolysaccharides of the biofilms produced by Gram-negative and Gram-positive bacteria [49]. The latter enzymatic treatment did not disrupt, to any significant degree, the biofilms formed by either encapsulated or unencapsulated bacteria (Figure 4A). In contrast, when preformed 48-h-old biofilms were treated with proteinase K, we noted a significant reduction in terms of CV staining (Figure 4B). We added proteinase K only to preformed mature biofilms in order to avoid proteolytic destruction of the Fg coating, which would have prevented early biofilm formation.

GBS expresses several surface proteins containing an LPXTG cell wall anchoring sequence. These cell wall proteins mostly function as adhesins by enabling GBS to bind to extracellular matrix components and epithelial cell receptors, while their involvement in GBS biofilm assembly received less attention. For these reasons, we tested a GBS mutant strain with non-functional Sortase A (Srt*A), an enzyme that is responsible for covalently anchoring the LPXT sequence of LPXTG-containing proteins to the cell wall. Absence of LPXT-linked cell wall proteins in the Srt*A mutant resulted in the almost complete abrogation of biofilm formation on hFg-coated surfaces in comparison with WT bacteria (Figure 4C). Taken together, these data show that matrix components of a protein nature, as well as cell-wall-anchored proteins play important roles on biofilm formation by GBS on hFg-coated surfaces.

### 3.4. Absence of SaeR Is Associated with Lower eDNA Levels in the Biofilm Matrix

**Figure 5:**
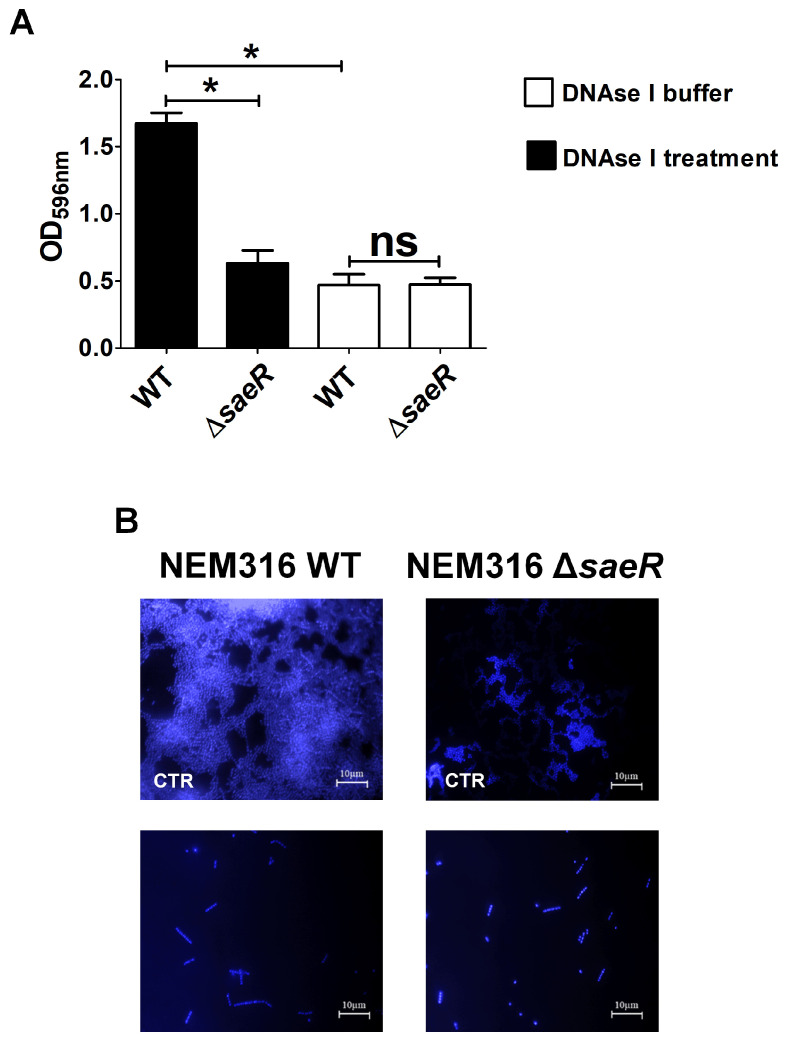
Role of eDNA on GBS formation on human fibrinogen. (**A**) Biofilms formed on human fibrinogen by wild-type (WT) and *saeR*-deleted mutant (Δ*saeR*) strains were incubated in presence of DNAse I (250 µg/mL) or its diluting buffer, used as control, for 2 h before staining with CV (CV). Bacterial biomass was quantified by measuring optical density at 596 nm (OD_596nm_). Results are means ± SD from three independent experiments performed in triplicate. ns, not significant; * *p* < 0.05, as determined by Mann–Whitney statistical analysis. (**B**) Fluorescence microscopy images of DNAse I-treated biofilms. CTR, biofilms treated with DNAse vehicle control. WT and Δ*saeR* 48-h-old biofilms were visualized with 4′,6-diamidino-2-phenylindole (DAPI, blue). Shown are representative images of three independent experiments. Scale bar = 10 µm.

Microscopic analysis was conducted by staining biofilms with 4′,6-diamidino-2-phenylindole (DAPI), a blue fluorescent stain that binds to both intracellular or extracellular double-stranded DNA (dsDNA). Using DAPI, we noted the presence of an amorphous blue staining material in mushroom-like elements of the biofilm, which was hypothesized to reflect the presence of eDNA. To confirm this, we treated 48-h-old WT and Δ*saeR* hFg biofilms with DNAase before CV staining and microscopic examination. In the WT biofilms, DNAse I digestion resulted in significant biofilm disruption, as evidenced by CV staining and, upon microscopic examination, in the disappearance of the amorphous halo that was, instead, still present in the biofilms treated with the DNAse I vehicle (Figure 5A,B). We also found that DNAse treatment resulted in reduced numbers of streptococcal cells in the biofilm (Figure 5B), suggesting that eDNA has a structural role in maintaining the organization of bacterial cells in a biofilm. Remarkably, DNAse I treatment had no effect on the levels of CV staining of Δ*saeR* hFg biofilms, and amorphous halos could not be detected in such biofilms either before or after DNAase treatment (Figure 5B).

All together, these results suggested that eDNA, likely released by dead bacteria, might represent a feature of well-developed and mature GBS biofilms. Moreover, eDNA might play an important role in the organization of the biofilm matrix and in promoting bacterial persistence within this structure.

## 4. Discussion

### 4.1. New Biofilm Formation Method

During the last few decades, considerable attention has been devoted to the ability of pathogens to form biofilms. After GBS biofilms were first detected in intrauterine devices [50], various attempts have been made to identify in vitro conditions that would resemble in vivo GBS biofilm formation. For example, GBS was grown in different laboratory media in both stationary and non-stationary conditions [31,33,46]. It was found that various bacterial factors, such as the genotype, the source (e.g., bovine vs. human; clinical isolates vs. isolates from asymptomatic carriers), and the expression of different virulence factors, can affect the ability of GBS to form biofilms [32,51]. In addition, various experimental conditions were found to influence biofilm formation, including the concentration of glucose and the pH of the growth medium, with sometimes contradictory results [31,32,52,53,54,55]. Most of the cited studies have been performed under static culture conditions (“batch mode”), which are unlikely to capture the interactions occurring in vivo between GBS and the host surfaces. Indeed, under these conditions, bacteria are known to be exposed to the potent action of shear forces generated by the flow of secretions over mucosal surfaces. To overcome this problem, D’Urzo et al. developed a method involving incubation under shaking (60 revolutions per minute), which prevents passive bacterial deposition [31]. Moreover, according to this method, the medium is changed after an 8 h incubation period (“fed batch mode”), resulting in increased numbers of live and metabolically active bacterial cells. However, despite these advancements, the precise environmental and bacteria conditions that are required for GBS biofilm formation are still presently unclear.

In the studies cited above, several abiotic surfaces have been tested. However, natural tissue surfaces and in vivo implanted medical devices are both characterized by the surface presence of ECM proteins, such as hFg, plasminogen, fibronectin and vitronectin that are recognized by various pathogens and thereby promote biofilm formation [56,57,58]. Colonization of host surfaces by GBS, in particular, is promoted by extracellular matrix components that are highly expressed on host surfaces.

Therefore, in the present study, we have developed a new method, based on considerations that in vivo biofilm formation only occurs after specific bacterial adherence to host receptor molecules, among which hFg is one of the most important in the case of GBS. In particular, we have modified the “fed batch” method of D’Urzo et al. by: (1) increasing the velocity of plate motion to 100 revolution per min; (2) coating the plate wells with hFg; (3) increasing the surface area by using 12-well instead of 96 well plates. The last modification was done since we noted higher non-specific deposition of bacterial cells along the line of contact between vertical and horizontal well surfaces using 96 well plates. We found in preliminary experiments that very few streptococcal cells adhere to non-coated plastic surfaces with shacking at velocities of 100 rpm or higher, unless the plates are coated with hFg. Notably, biofilm development and maturation are considerably increased using the newly developed method in comparison with the traditional static culture method, thereby allowing the analysis of the molecular requirements for biofilm formation. One of such requirements involves the presence of functional sortase A, which is required to covalently link cell wall proteins to the bacterial peptidoglycan backbone. This requirement was evidenced here by the inability of a Srt*A mutant to assemble biofilms on hFg-coated surfaces. These data are at variance with a previous study using static culture conditions [48], highlighting again the differences between our method and the previously used ones.

### 4.2. Role of SaeRS in Biofilm Formation

Recent data have evidenced a major role of TCSs in bacterial pathogenesis, making these systems interesting targets for the development of alternative strategies to control antibiotic-resistant infections. Considerable attention has been devoted to the mechanisms underlying biofilm formation by *S. aureus* and other staphylococci, which frequently induce serious infections by colonizing implanted medical devices. One of the regulatory systems used by staphylococci to modulate biofilm formation is the SaeRS TCS, which is highly homologous to a similar system in GBS [37]. The *S. aureus* Newman strain expresses a constitutively activated SaeRS system that inhibits biofilm formation [59]. Moreover, *SaeRS* deletion in *S. epidermidis* resulted in increased biofilm formation, which was linked to increased bacterial autolysis and the secondary release of eDNA, the latter playing an important role in the organization of the biofilm extracellular matrix [43].

In view of the ability of the SaeRS system to regulate biofilm formation in staphylococci, we studied the role of the homologous system in GBS. We found here that the deletion of the SaeR response regulator results in a form of sessile GBS growth that lacks several features of a well-developed, mature biofilm, such as vertical growth, structured, multilayered arrangement of bacterial cells and eDNA. These data raise the possibility that the inability of GBS to form abundant biofilms in the absence of SaeRS is linked to defective bacterial autolysis and eDNA release. This hypothesis is in line with the observations on the essential role of eDNA, particularly nuclease-resistant Z-DNA, in biofilm organization [60]. Alternatively, the scarcity of eDNA in the absence of SaeRS might simply reflect a reduction in the number of streptococcal cells and/or a lack of biofilm maturation. For these reasons, studies are underway to determine whether SaeRS has a role in inducing autolysis and the release of eDNA during planktonic and sessile growth. Moreover, in view of the regulatory role of SaeRS, it is possible that the effects observed here are secondary to the SaeRS-dependent upregulation of the molecules that promote GBS biofilm formation. Since SaeRS was recently shown to positively regulate the expression of a restricted number of genes comprising those encoding for the PbsP and BvaP adhesins and for SaeRS [37,39,40], we are currently examining the role of these adhesins in biofilm formation. Moreover, recent data indicate that SaeRS does not play a significant role in the regulation of gene expression in the NEM316 strain used here during planktonic growth [39]. For this reason, it will be of interest to examine the differential gene expression in GBS during sessile growth in the presence and in the absence of the SaeRS system. Moreover, given the increasing interest in the potential role of eDNA and other bacterial nucleic acids as pathogen-associated molecular patterns [61,62,63,64,65,66], studies are underway to identify the GBS biofilm components capable of stimulating innate immune responses and their cognate pathogen recognition receptors. Such studies might be useful in developing new host-directed strategies aimed at stimulating innate immune responses to treat infections by GBS and other pathogens, as recently proposed [61,62,63,64,65].

### 4.3. Limitations of the Present Study and Conclusions

A major limitation of the present study is that the mechanisms underlying the ability of SaeRS to promote biofilm formation were incompletely investigated. Gene regulation by this TCS have been previously analyzed under conditions of plantonic growth, and little is known of the role of this signaling system during sessile growth and biofilm formation. It is, therefore, expected that major insights will come from transcriptomic biofilm analyses using strains harboring mutations in SaeRS and other TCSs. Studies are underway to analyze these points.

In conclusion, we show here that, in contrast to abiotic supports, hFg-coated surfaces strongly promote biofilm formation by GBS as structured masses in a dense matrix, and this may represent a useful model for studying host–pathogen interactions. Furthermore, in the present study, it was possible to identify a major role of the two-component regulatory system SaeRS in biofilm formation. These data could be useful for clarifying the mechanisms underlying interactions between GBS and the human host in order to develop new molecular strategies to control colonization and infection as alternatives to antibiotic treatment.

## Data Availability

The original contributions presented in the study are included in the article/Supplementary Materials, further inquiries can be directed to the corresponding author.

## Supplementary Materials

The following supporting information can be downloaded at: https://www.mdpi.com/article/10.3390/microorganisms12102096/s1, Figure S1: Biofilm formation on hFg is a GBS property; Figure S2: GBS biofilm formation in static growth conditions; Figure S3: GBS wild-type and mutant strains growth curves.
