# Supplementary material for: Role of the SaeRS Two-Component Regulatory System in Group B Streptococcus Biofilm Formation on Human Fibrinogen
Source: Microorganisms. 2024 Oct 20;12(10):2096. doi: 10.3390/microorganisms12102096 (PMC11510217; doi:10.3390/microorganisms12102096)
Supplement: Supplementary file 1 [file microorganisms-12-02096-s001.zip › microorganisms-3253092-supplementary.pdf]

Supplementary Figures and legends:

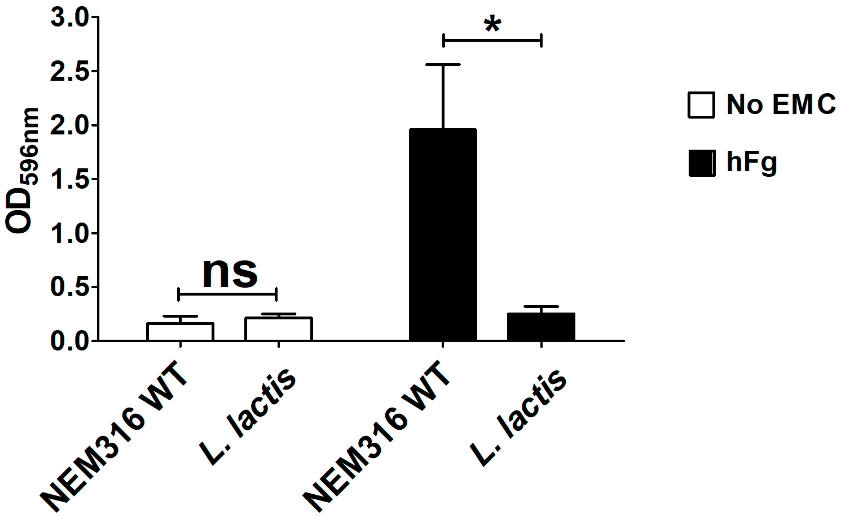

Figure S1. Biofilm formation on hFg is a GBS property. Biofilm formation abilities of GBS NEM316 wild-type (WT) and *Lactococcus lactis* subsp. *Cremoris* MG1363 strains on twelve-well plates coated with human fibrinogen (hFg) or uncoated wells (no EMC). GBS and *L. lactis* biofilms were analyzed at 48 h for crystal violet (CV) staining (OD_{595nm}). Results are means ± SD from three independent experiments performed in triplicate. Ns, not significant; *, p<0.05, as determined by Mann-Whitney statistical analysis.

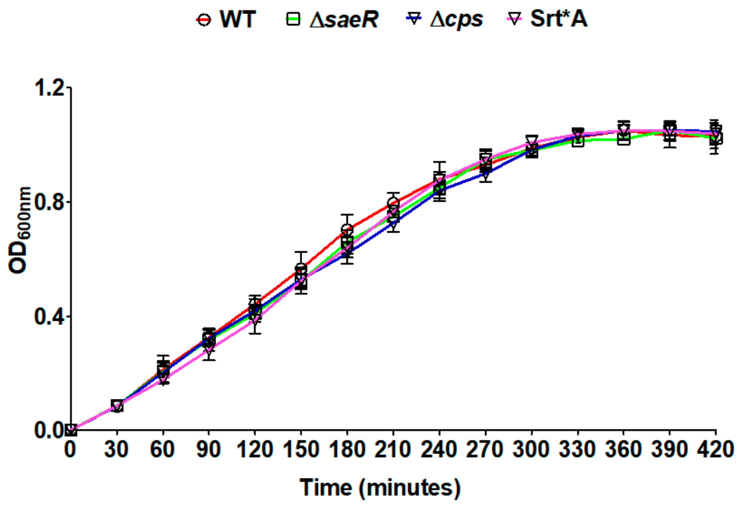

Figure S2. GBS biofilm formation in static growth conditions. Biofilm formation abilities of GBS NEM316 wild-type (WT) and NEM316 strains with mutations in *saeR*, *cps* and *srtA* (Δ*saeR*; Δ*cps* and Srt*A) and *Lactococcus lactis* subsp. *Cremoris* MG1363 strain on twelve-well plates coated with human fibrinogen (hFg) or uncoated wells (no EMC). Bacterial growth was analyzed at 48 h for crystal violet (CV) staining (OD_{595nm}). Results are means ± SD from three independent experiments performed in triplicate.

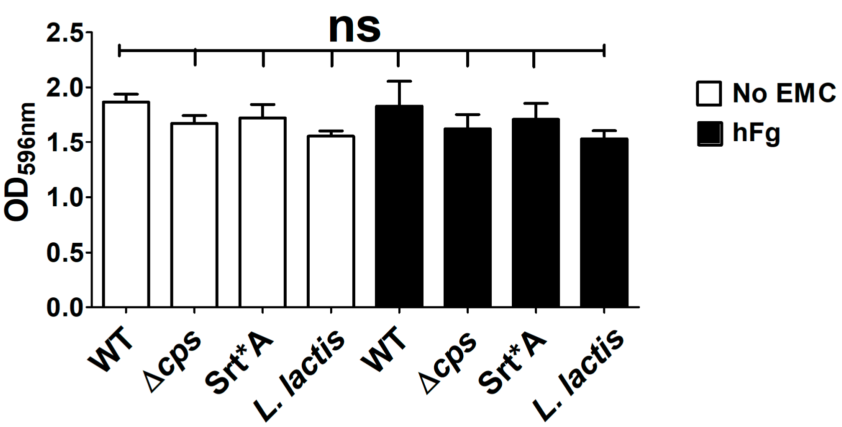

Figure S3. GBS wild-type and mutant strains growth curves. Shown are representative growth curves of the following GBS strains: NEM316 wild-type (WT), mutants deleted for *saeR* or *cps* (Δ*saeR* or Δ*cps*, respectively) or with non-functional SrtA (Srt*A). Bacteria were grown in Todd-Heiwitt-Broth supplemented with 0.5% extract yeast (THY) and optical density was measured (OD_{600nm}) at the indicated times. Ns, not significant; as determined by Mann–Whitney statistical analysis.
